# Effect of Recycling on the Mechanical, Thermal and Rheological Properties of Polypropylene/Carbon Nanotube Composites

**DOI:** 10.3390/polym14235257

**Published:** 2022-12-01

**Authors:** Attila Bata, Dorottya Nagy, Zoltán Weltsch

**Affiliations:** Department of Innovative Vehicles and Materials, GAMF Faculty of Engineering and Computer Science, John von Neumann University, 6000 Kecskemét, Hungary

**Keywords:** copolymer, polypropylene, MWCNT, nanocomposites, rheological properties, recycled MWCNT

## Abstract

In this research the effect of physical recycling on the mechanical, thermal, and rheological properties of polypropylene (PP)/multiwalled carbon nanotube (MWCNT) was investigated. After melt homogenization by extrusion, specimens were injection moulded with 0.1 and 0.5 wt% MWCNT content. The recycling process was simulated by multiple grinding and re-moulding, then we compared the behavior of original and recycled PP/MWCNT composites. Differential scanning calorimetry (DSC) measurements proved that MWCNT had double the effect on the morphology of the PP matrix: on the one hand nucleating effect can be detected because 0.5 wt% MWCNT increased the onset temperature of crystallization by 10 °C, compared to the basic PP material; on the other hand, the crystalline fraction of the recycled composite materials decreased compared to the original PP material with the same MWCNT content. This resulted in a slight decrease in strength and stiffness but an increase in elongation at break. However, compared to the original unreinforced PP reference, even the recycled materials have better properties. The mechanical test results showed that recycled PP/MWCNT 0.5 wt% increased the elastic modulus (~15%) and decreased the tensile strain at yield (~10%). However, in the values of tensile stress at yield, relevant difference was not found. It was also shown by oscillatory rheometry that MWCNT had a significant effect on the rheological properties (storage and loss modulus, complex viscosity) of PP compounds in a wide temperature range (190–230 °C).

## 1. Introduction

Nowadays polypropylene (PP) is one of the most common plastics because of its excellent properties, such as chemical resistance, easy processing, lightness, etc. Ethylene-propylene block copolymer (ethylene-propylene block copolymer with a low content of ethylene) is a semi-crystalline polymer with a higher flexibility and a higher impact strength than polypropylene homopolymer [1,2,3,4,5]. PPCP is mainly used for blow molding, injection molding and sheet extrusion applications, where high clarity is required. These materials are used for food and medical packaging and consumer products [5]. However, unreinforced polymers possess some disadvantages in terms of their mechanical and thermal properties [6]. Since Iijima discovered the structure of carbon nanotubes (CNTs) [7,8] many advanced properties of CNTs have been explored, such as physical, electrical, mechanical, thermal and magnetic properties [9,10,11,12,13]. The electrical conductivity of polymers can be adjusted with various CNT contents. CNT/polymer blends can be used as antistatic materials, electrostatic discharge (ESD) material or as shielding materials for electromagnetic interference (EMI) or radio frequency interference (RFI) [14]. Unmodified CNTs may exhibit agglomerate phenomenon and poor interfacial adhesion, hence it is difficult to disperse MWCNT in polymer matrix uniformly [7]. Several studies have reported the influence of MWCNT on the rheological and mechanical behavior of PP nanocomposites. Xu et al. studied the effect of carbon nanotube ratio on the rheological properties (shear rates are 1–102 s^−1^) of isotactic polypropylene/MWCNT composites. They found that the aspect ratio of MWCNT showed a strong influence on MWCNT gelation concentration [15].

Salih Hakan Yetgin investigated MWCNT filled PP nanocomposites and found that storage and loss modulus, complex viscosity and relaxation time of the neat polymer increased by adding MWCNT, which makes this material ideal for high shear rate processing with improved long-term time dependent physical properties. This behavior in the linear viscoelastic regime can be explained by the increasing interactions between nanotube–nanotube and polymer–nanotube [6,16]. Several literature sources reported that the nanotube–nanotube interactions are responsible for the electrical percolation and the polymer–nanotube interactions are responsible for the rheological percolation [17,18]. That way a network-like structure will form from the MWCNT, which hinders the segmental motion of the polymer chains [19]. Prashantha et al. reported a similar result for maleic anhydride (MA) compatibilized PP [20]. At a low frequency range, a transition liquid to solid-like behavior was observed with 2 wt% of MWCNT. The percolation threshold (nanotube network) can also explain this behavior. Using storage modulus values at low frequencies, several parameters (e.g., formation of percolation threshold or polymer–filler interactions) could be determined by mathematical methods [18,21,22]. In a study by Verma et al., storage and loss modulus also increase with MWCNT; the composites show terminal behavior up to 0.4 wt% MWCNT, then the dependence of modulus on frequency weakens. Frequency dependence also shows that adding CNT does not change the short-range motion of the polymer, but it affects the long-range motion [21,23]. Therefore, MWCNT does not affect the glass transition temperature.

Several studies investigated PP/MWCNT nanocomposites by rheological, thermal, and mechanical methods, but just a few determined zero-shear-rate viscosity in a wide temperature range, melting and crystallization behaviors, and mechanical properties of recycled PP/MWCNT nanocomposites. In a number of manufacturing technology areas, the re-use of recycled raw materials is increasingly demanded [24]. Recycled PP nanocomposites may be suitable for multiple uses, without significantly increasing the environmental impact of their production and can also contribute to the development of a circular economy. There are few results available about the investigation of recycled PP nanocomposites in the literature. In our study, we manufactured 0.1–0.5 wt% PP/MWCNT, then recycled them. Standard specimens were injection molded, then they were grounded by a grinder. This process was repeated five times to reach five-times recycled materials. Before the rheological, thermal, and mechanical investigation, the recycled materials were regranulated to get the same form as the original material.

## 2. Materials and Methods

TIPPLEN K499 type polypropylene by MOL Petrochemicals was used; K499 is an ethylene-propylene impact block copolymer for injection molding containing 5–7% ethylene co-monomer [11]. The melt flow index (MFI) was 6.5 g/10 min at 230 °C and 2.16 kg. Specific gravity was 0.9 g/cm^3^ [11].

PLASTICYL TM (Nanocyl SA) is a family of multiwall carbon nanotubes (MWCNTs) thermoplastic concentrates for applications requiring superior electrical conductivity and electrostatic discharge (ESD) properties [12]. PLASTICYL TM PP2001 is a conductive masterbatch based on polypropylene loaded with 20% of Nanocyl’s MWCNTs (NC7000™). Because of its low viscosity and high flow formulation, PLASTICYL TM PP2001 is ideal for injection molding and extrusion processes [12].

Composites containing 0.1 wt% and 0.5 wt% MWCNT were prepared in two compounding steps. The mixing process was performed by a Scientific Labtech 26 mm diameter twin screw extruder with a standard 32 L/D ratio [13]. The screw speed was 330 rpm. In order to reach homogenization, the first mixing was performed at a barrel temperature of 170–190 °C, then the second mixing at a higher temperature (190–230 °C).

Scanning electron microscopic images (SEM) of the composites were recorded. During the preparation of the samples a thin layer of gold was applied by sputtering. A SEM image was taken by Hitachi S300N in a high vacuum on 20 keV [25].

Standard specimens (ASTM D638) were injection molded by a Wittmann Battenfeld Ecopower 55–130 type machine [26], then they were ground by a grinder. This process was repeated five times to reach five-times recycled materials. Before the rheological, thermal, and mechanical investigation, the recycled materials were regranulated to get the same surface/volume ratio as the original material.

Mechanical properties of standard specimens were measured with INSTRON 3366 universal test machine. The crosshead speed was 1 mm/min until 0.3% strain, then 5 mm/min according to the ASTM D638 standard [27,28].

Melting and crystallization behavior of PP nanocomposites were measured by differential scanning calorimetry (DSC) (TA Instruments, Q200) [29]. The DSC was used to investigate the crystallinity (*X_c_*) of the PP/MWCNT composites. Crystallinities were calculated by using the following equation [Equation (1)] [30].

(1) $$X_{c}=\frac{\Delta H_{m}}{\Delta H_{m}^{0}}*100\;\left[\%\right]$$

where ∆*H_m_* is the melting enthalpy value of sample measured in the second heating cycle (J/g), $\Delta H_{m}^{0}$ is the thermodynamic melting enthalpy value of PP having 100% of crystallinity (207 J/g) [30]. The sample was heated by two stages in a nitrogen environment. First, the sample was heated from 30 to 200 °C at a heating rate of 5 °C/min and kept at this temperature for 1 min (remove the thermal history), then it was cooled from 200 to 30 °C with the cooling rate of 5 °C/min to release the internal stress of the sample. The second heating cycle was also from 30 to 200 °C at 5 °C/min.

The rheological investigation was performed by a TA Ares G2 (TA Instruments, Inc., New Castle, DE, USA) type oscillation rheometer with 25 mm diameter cone-plate geometry at different temperatures (190 °C, 210 °C, 230 °C) [29]. The materials were measured in 0.05–300 rad/s angular frequency with 5% deformation amplitude. Each measurement was carried out three times at each parameter set. The zero-shear-rate viscosity values were determined from the complex viscosity vs. angular frequency curves using Carreau–Yasuda model by TRIOS software for the extrapolation [29]. The Van Gurp–Palmen plot, which presents the phase angle as the function of the complex modulus, can be used to investigate the elastic and/or viscous dominance [31,32]. The Cole–Cole plots, which show the connection of loss and storage modulus, are indicative of the heterogeneity of the composite [33,34]. Imperfect circles suggest inhomogenity of the system. Another technique to obtain information about the homogenization is using the Cox–Merz empirical law, which is applicable to most linear polymers. The viscosity curve as the function of shear rate is similar to the complex viscosity curve as the function of angular frequency Equation (2) on [35]. This equation states that the measurements by oscillation are in accordance with the rotational studies. The Cox–Merz rule is not valid for polymer melts containing large amounts of CNTs because the shear viscosity is usually higher than the complex viscosity [36]. However, when the CNT content is low and the composites are below the percolation threshold, it can be used [37,38].

The oscillation rheometer measures at a low shear rate range, so it is appropriate for determining the zero-shear-rate viscosity. The early period of the viscosity-shear rate curve is generally extrapolated by Carreau–Yasuda model for polymers, sufficiently good for the viscous flow behavior in the region of the very small as well as the very large shear rates (Equation (3) [39]).

(2) $$\eta (\overset{\cdot }{\gamma })=\eta *(\omega ),$$

(3) $$\frac{\eta -\eta_{\infty }}{\eta_{0}-\eta_{\infty }}=\frac{1}{{\left[1+{\left(t_{1}\overset{\cdot }{\gamma }\right)}^{a}\right]}^{\frac{1-n}{a}}}$$

where [*t*_1_] is a time-featured quantity, a parameter that characterizes the transition between zero-shear-rate viscosity and the measured part of the viscosity curve, [*n*] exponent is known from the power law. The [*a*] transition parameter, [*η*_0_] the zero shear rate viscosity, [*η*_∞_] the infinite viscosity at the infinite shear rate, [*γ*] effective shear rate. The t_1_ characteristic time and a parameter can be related to the molecular weight (*M_w_*) value and the polydispersity, respectively [40]. The main advantage of Carreau–Yasuda model is that the viscosity at zero-shear rate can be determined.

## 3. Results

**Figure 1:**
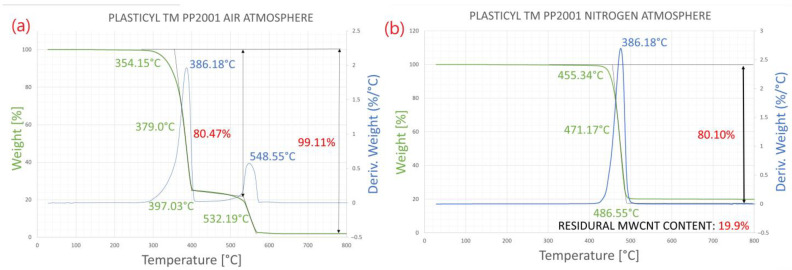
TG curve of PP/MWCNT masterbatch in air (**a**), and nitrogen (**b**) atmosphere.

The MWCNT content of the PP masterbatch was verified by thermogravimetric analysis (TA Q5000) [29,41] in nitrogen atmosphere and air (Figure 1). All experiments equally showed ~20 wt% MWCNT content. Figure 1a shows that a two-step decomposition is observed, where the PP matrix material of the masterbatch and the MWCNT start to decompose at different temperatures. When measured in an inert gas (Figure 1b), it can be seen that the MWCNT has not decomposed, and the residual filler content is ~20 wt%.

**Figure 2:**
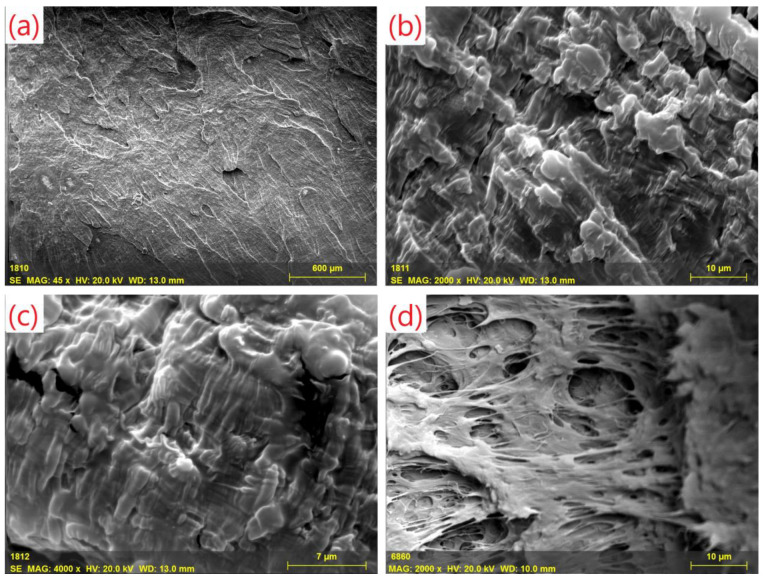
SEM images of (**a**) PP polymer, (**b**,**c**) 0.5 wt% filled PP/MWCNT nanocomposites, (**d**) PP masterbatch.

Homogeneous distribution of nanofillers in PP matrix is crucial to produce nanocomposites having the same rheological properties. Electron micrographs show detailed images of the material’s morphological characteristics. The strengthening effect of MWCNT is highly dependent on the amount of nanotubes, as well as the degree of distribution in the matrix material. With the sample preparation method used, no agglomerates can be detected in the images. Proper homogenization of MWCNT was also validated by oscillatory rheological measurements. SEM images of PP polymer, and PP filled with 0.5 wt% MWCNT can be seen in Figure 2.

Results of mechanical properties, such as elastic modulus or tensile stress at yield, can be attributed to several factors according to the literature. Increase in elastic modulus can be associated with the stiffness properties of MWCNT. The rigidity of MWCNT decreases the mobility of PP chains, which results in lower flexibility and higher strength. In his study, Dimitrios [42] reports that the modulus and the tensile strength of PP/MWCNT increased with the addition of 2–2.5 wt% MWCNT. Low MWCNT content (2–2.5%) acts as a reinforcing material, however a higher amount causes agglomeration. CNT dispersed well in the polymer matrix, resulting in greater load distribution in the structure of composite. Strong interactions between polymer chains and CNT provide more efficient load transfer to nanotubes [42,43,44]. Table 1 summarizes the results of mechanical properties (based on tensile tests).

The modulus value of PP raised from 999 MPa to 1226 MPa at 0.5 wt% MWCNT content, and as for the recyclate of this composite, it changed to 1156 MPa. The increase of modulus in the case of the recycled 0.5 wt% MWCNT nanocomposite is 15.7% compared to the original PP matrix. According to the results, modulus and strength values of recycled nanocomposites outperform the values of original unreinforced PP material.

Presumably multiple recycling does not significantly break the MWCNT, and thanks to the better dispersion and adhesion it enhances the reinforcing effect. It is probable that the fracture of polymer chains during recycling has a higher impact on the results. Regarding the values of tensile stress at yield, relevant differences were not noted. However, the addition of MWCNT obviously reduced the tensile strain at yield, which was indicated by the modulus values, as well. Presumably, the changes to nodes of physical spatial network are limited by MWCNT, which could cause the decrease of tensile strain at yield.

Literature research showed that MWCNT helps the crystallization of PP [6]. The degree of crystallinity is also linked to the fact that compatibilizers dramatically improve the interphase adhesion between surface of the CNT and the polymer chains; this way the reduction of matrix polymer’s segmental mobility causes nodule formation [6]. There are several studies researching the effect of MWCNT on the crystallinity of PP [6]. In these studies, they found that the degree of crystallinity of PP can stay unchanged; it can decrease or slightly increase with a higher MWCNT content. For example, Salih Hakan Yetgin’s research indicates that the *X_c_* in nanocomposites changes depending on the amount of MWCNT. *X_c_* value of PP polymer increased by 5% at 0.3 wt% MWCNT, but with the addition of more MWCNT that value decreased [6].

**Figure 3:**
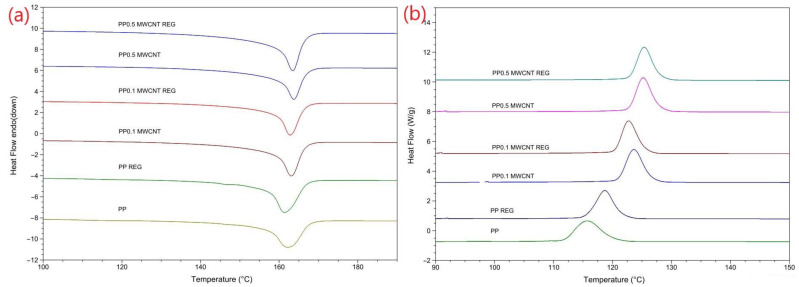
DSC curves of melting endotherms (**a**) and exoterms (**b**) of PP/MWCNT nanocomposites.

The melting temperature of PP polymer, its nanocomposites, and their recyclate ranged between 162–164 °C. This is a perfectly adequate melting result for a standard PP block copolymer. The crystal melting curves show that the peaks, with the addition of MWCNT gets narrower, presumably because of the formation of different sized crystals. It is most likely that the structure of the nanotubes hinder the original size of the crystals (Figure 3a).

One can also see that with the addition of MWCNT, the amount of crystals increases (Figure 3a). This phenomenon can also be seen in the case of recycled MWCNT composites. The *X_c_* value of nanocomposites changed depending on the number of recycling steps and the MWCNT content. Recycled PP/MWCNT composites show almost the same crystallinity values as the PP polymer. As it is known, the ratio of the crystalline fraction can be linked to the mechanical properties. In our case, the change in crystalline structure (crystal size, quantity) can affect the mechanical properties in the positive direction (modulus of elasticity). PP/MWCNT composites with 0.5 wt% MWCNT reinforcement, as in many studies [6], increased the crystallinity values by 4% compared to the original PP. (Table 2).

Effects of MWCNT in the case of the crystallization behavior of polymers were studied in a wide temperature range [6,42,45,46]. All of them stated the nucleating effect of CNT, which provides easier and faster crystallization under isotherm and non-isotherm circumstances. Because of the very similar crystallinity and multiple nucleation place, the expected spherulite size of composites filled with nanotubes will be much smaller than that of unreinforced PP. It was found that the melting behavior and melting temperature of PP (T_pm_) did not change significantly with the addition of MWCNT [6,42,45,46].

Comparing the original and recycled material pairs, it can be seen that the recycled nanocomposites with 0.1 wt% and 0.5 wt% CNT content have a lower crystalline fraction compared to the original unreinforced PP material. The smaller crystallite fraction in the recycled composites is most likely due to further homogenization caused by the five consecutive grinding and injection moulding and the more dispersed nanotubes interfere/obstruct the formation of larger crystallites/spherulites. This lower fraction may explain the higher elongation at break and the lower strength/stiffness of the recycled composites compared to the original unreinforced material.

Interpretation of Table 2:

∆*H_m_*, melting enthalpy; T_pm_, melting temperature; *X_c_*, degree of crystallinity; ∆H_c_ crystallization enthalpy; T_pc_, crystallization peak onset temperature; T_eic_, crystallization peak temperature.

On the crystallization curves, one can observe (Figure 3b) that the recycled PP/MWCNT composites, compared to the original PP, has an increased onset temperature by 7–10 °C and an increased maximum temperature by 5–8 °C depending on the volume of MWCNT. We can state that the maximum temperatures of recycled PP/MWCNT composites are the same; the crystallization onset temperatures also show minimal differences compared to the PP/MWCNT composites. The regranulate of PP polymer shows a 3 °C crystallization onset temperature difference compared to that of PP polymer (Table 2).

**Figure 4:**
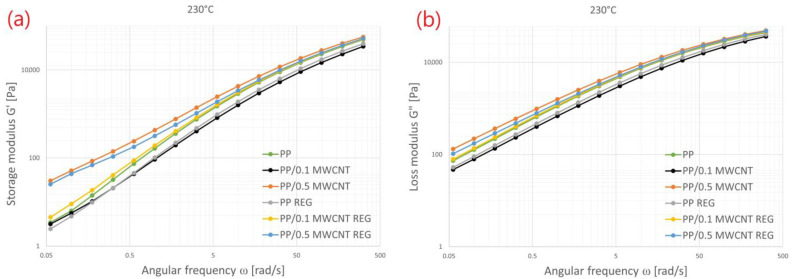
Measured curves ((**a**) storage and (**b**) loss modulus) of the PP/MWCNT by oscillation at 230 °C.

The viscoelastic properties of PP/MWCNT nanocomposites at 230 °C is shown in Figure 4a,b. We can see in the Figures that the storage and loss modulus of PP/MWCNT increased significantly in the case of a 0.5 wt% MWCNT addition compared to the original PP, particularly in a low frequency range. This behavior was supported by many studies, for example, Salih Hakan Yetgin extensively studied the rheological behavior of PP nanocomposites with different MWCNT contents [6].

The difference in storage modulus (G’) is more relevant in the low frequency range. This behavior is the natural reaction of thermoplastic composites in LVE (linear viscoelastic regime) that mostly leads to solid-state-like or pseudo solid-state-like behavior [6]. This can be explained by increasing MWCNT content as the nanotube–nanotube and polymer–nanotube interactions increased [21].

Figure 4a shows that the recycled PP/MWCNT 0.5 wt% composite, despite the recycling process, has a much higher storage modulus value than the original, unreinforced PP. It can be found that recycled PP/MWCNT composites keep their reinforcing effect in a low frequency range, especially in the case of a 0.5 wt% filling. Decrease of storage modulus (G’) in the case of recycled PP/MWCNT composite is presumably caused by the chain fragmentation of PP, recycling has less effect on the structure of the MWCNT.

**Figure 5:**
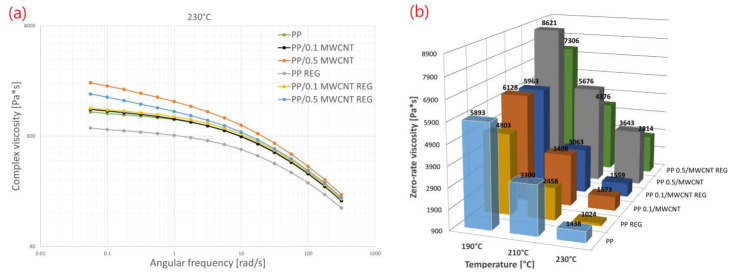
(**a**) Complex viscosity curves (230 °C); (**b**) zero-shear-rate viscosity in the function of the temperature of PP/MWCNT.

Zero-shear-rate viscosity was determined from dynamic measurements extrapolated by Carreau–Yasuda model [39]. The reference material was PP polymer. The measured complex viscosities in addition to storage and loss modulus curves as the function of angular frequency are shown in Figure 5.

According to the results it can be stated that in the low frequency range, MWCNT has a reinforcing and bracing effect in wide temperature interval (190–230 °C), but in the higher frequency range (at higher deformation speed) viscosity differences decreased significantly between PP/MWCNT composites before and after recycling. This also proves that during injection molding, we can produce standard PP/MWCNT test specimens without changing the technological parameters. We did not experience viscosity change during the injection molding technology process (e.g., burr, partially filled piece). Similar viscosities appearing in higher deformation speed according to certain studies can be linked to chain slippage [5].

Zero-shear-rate viscosity values for all materials (PP reference polymer, PP/MWCNT, PP/MWCNT REG) are shown in Table 3.

In Table 3, the zero-shear-rate viscosity values of the materials can be seen at three different temperatures. The values were calculated from three measurements at the same parameters. The relative standard deviation values’ average is under 5%. From the results of deviation, we can state that the addition of MWCNT into the PP matrix is adequate.

It can also be stated that the zero-shear-rate values of recycled PP/MWCNT composite (0.5 wt%) in a wide temperature range are higher than the standard PP polymer. The zero-shear-rate viscosity value of PP/MWCNT 0.5 wt% REG at a relatively high polymer-melt temperature (230 °C) is almost 96% higher than that of original PP.

## 4. Conclusions

In our study, we produced PP/MWCNT nanocomposites containing different quantities of nanotubes (0.1; 0.5 wt%). For compounding, we used twin-screw extruder and produced standard polymer samples with injection molding. Mechanical, rheological, and thermal properties of the composites were studied before and after a recycling process, which consists of multiple grinding and injection moulding.

It was found that the tensile modulus in the case of recycled PP/MWCNT 0.5 wt% composite increased by 15% compared to our reference (original, unreinforced) PP polymer, but as an effect of the recycling, its modulus decreased by 7% compared to the PP/MWCNT 0.5 wt%. Tensile strain at yield did not show significant difference.

Thermodynamical experiments showed that the onset temperature of crystallization of the recycled PP/MWCNT 0.5 wt% composite significantly increased (by 10 °C) compared to the reference PP material. The maximum temperature of crystallization decreased by 8 °C. In the melting temperatures, significant differences were not found; they differed between 162–164 °C.

Considering the rheological investigations, we can say that the distribution of MWCNT in the PP matrix is homogeneous. The deviation value of multiple simultaneous experiments, and results measured at different temperatures (190; 210; 230 °C) are ~5%, or below that. In addition, considering the results, we can see that the zero-shear-rate viscosity values of recycled PP/MWCNT composite (0.5 wt%) in a wide temperature range are higher than the reference PP polymer. The zero-shear-rate viscosity value of recycled PP/MWCNT 0.5 wt% composite at a relatively high temperature (230 °C) is nearly 96% higher than the original, unreinforced PP polymer. It is most likely that MWCNT creates a physical grid in the PP matrix. This may explain the increase in viscosity and modulus. In the case of regranulation, an increase in interaction may be the cause. This can also explain the nucleating effect (change in surface energy).

Considering the results, we can conclude that the MWCNT, has reinforcing effect even after multiple recycling steps, which can make the PP more suitable for a wide range of technical applications.

## Figures and Tables

**Table 1:** Results of mechanical properties (tensile) of PP and PP/MWCNT nanocomposites (negative changes highlighted in red, positive changes in green).

*Sample*	Young Modulus (MPa)				Tensile Stress at Yield (Mpa)				Tensile Strain at Yield (%)			
	Young Modulus (MPa)	Std. dev (MPa)	Relative std. dev (%)	Difference from the Unreinforced Original PP (%)	Tensile Stress at Yield (Mpa)	Std. dev (MPa)	Relative std. dev (%)	Difference from the Unreinforced Original PP (%)	Tensile Strain at Yield (%)	Std. dev (%)	Relative std. dev (%)	Difference from the Unreinforced Original PP (%)
PP	**999**	3.9	0.39	-	**22.12**	0.23	1.03	-	**5.33**	0.09	1.75	-
PP REG	**958**	9.1	0.95	−4.1	**21.44**	0.15	0.71	−3.1	**5.47**	0.09	1.70	2.6
PP 0.1/MWCNT	**1174**	16.1	1.37	17.5	**22.56**	0.06	0.24	1.9	**4.52**	0.03	0.69	−15.2
PP 0.1/MWCNT REG	**1041**	61.6	5.92	4.2	**22.50**	0.19	0.83	1.7	**5.20**	0.36	6.92	−2.5
PP 0.5/MWCNT	**1226**	12.3	1.00	22.7	**22.92**	0.08	0.37	3.6	**4.50**	0.06	1.39	−15.5
PP 0.5/MWCNT REG	**1156**	67.1	5.81	15.7	**22.44**	0.23	1.03	1.4	**4.75**	0.37	7.73	−10.9

**Table 2:** Results of thermal properties (tensile) of PP and PP/MWCNT nanocomposites.

*Sample*	Second Heating			Cooling		
	∆*H_m_* (J/g)	T_pm_ (°C)	*X_c_* (%)	∆Hc (J/g)	T_pc_ (°C)	T_eic_ (°C)
PP	99.40	162.2	47.3	90.0	120.1	115.7
PP REG	100.80	162.4	48.2	89.2	121.6	118.7
PP 0.1/MWCNT	110.70	163.7	52.9	102.4	126.6	123.6
PP 0.1/MWCNT REG	99.50	163.2	47.6	91.4	125.6	122.7
PP 0.5/MWCNT	107.70	163.8	51.5	98.2	127.9	125.2
PP 0.5/MWCNT REG	101.40	164.0	48.5	92.7	128.0	125.3

**Table 3:** Results of zero-shear-rate viscosity of wide temperature range of PP/MWCNT (negative changes highlighted in red, positive changes in green).

*Sample*	190 °C				210 °C				230 °C			
	Zero-Shear-Rate Viscosity (Pa*s)	Std. dev (Pa*s)	Relative std. dev (%)	Difference from the Original Zero-Shear-Rate Viscosity (%)	Zero-Shear-Rate Viscosity (Pa*s)	Std. dev (Pa*s)	Relative std. dev (%)	Difference from the Original Zero-Shear-Rate Viscosity (%)	Zero-Shear-Rate Viscosity (Pa*s)	Std. dev (Pa*s)	Relative std. dev (%)	Difference from the Original Zero-Shear-Rate Viscosity (%)
PP	**5893**	234.2	3.97	-	**3300**	83.8	2.54	-	**1438**	35.4	2.46	-
PP 0.1/MWCNT	**6128**	172.9	2.82	+3.98	**3408**	35.8	1.05	+3.27	**1573**	45.6	2.90	+9.38
PP 0.5/MWCNT	**8621**	109.5	1.27	+46.29	**5676**	193.9	3.42	+72	**3643**	48.3	1.32	+153.33
PP REG	**4803**	4,6	0.10	−18.5	**2458**	91.1	3.71	−25.52	**1024**	59.5	5.81	−28.79
PP 0.1/MWCNT REG	**5963**	100.4	1.68	+1.18	**3063**	84.5	2.76	−7.19	**1559**	27.9	1.79	+8.41
PP 0.5/MWCNT REG	**7306**	15.0	0.21	+23.97	**4376**	3408.0	0.62	+32.6	**2814**	104.6	3.72	+95.68
